# *BRCA1* Promoter Methylation Status in 1031 Primary Breast Cancers Predicts Favorable Outcomes Following Chemotherapy

**DOI:** 10.1093/jncics/pkz100

**Published:** 2019-12-11

**Authors:** Olafur A Stefansson, Holmfridur Hilmarsdottir, Kristrun Olafsdottir, Laufey Tryggvadottir, Asgerdur Sverrisdottir, Oskar T Johannsson, Jon G Jonasson, Jorunn E Eyfjord, Stefan Sigurdsson

**Affiliations:** 1 Faculty of Medicine, University of Iceland, Reykjavik, Iceland; 2 Cancer Research Laboratory, Biomedical Center, University of Iceland, Reykjavik, Iceland; 3 Current affiliation deCODE genetics/Amgen Inc., Sturlugata 8, Reykjavik, Iceland; 4 Icelandic Cancer Registry, Icelandic Cancer Society, Reykjavik, Iceland; 5 Department of Oncology, Landspitali University Hospital, Reykjavik, Iceland; 6 Department of Pathology, Landspitali University Hospital, Reykjavik, Iceland

## Abstract

**Background:**

Breast Cancer 1 gene (*BRCA1*) is known to be inactivated in breast tumors by promoter methylation. Tumor cells in patients carrying a germline mutation in *BRCA1* are sensitive to cytotoxic drugs that cause DNA double strand breaks. However, very little is known on whether patients with *BRCA1* promoter methylated tumors are similarly sensitive to cytotoxic drugs. In this study, we address this by making use of extensive follow-up data on patients treated with cyclophosphamide, methotrexate, and fluorouracil in Iceland between 1976 and 2007.

**Methods:**

We analyzed *BRCA1* promoter methylation by pyrosequencing DNA from tumor samples from 1031 patients with primary breast cancer. Of those, 965 were sporadic cases, 61 were *BRCA2*, and five were *BRCA1* germline mutation carriers. All cases were examined with respect to clinicopathological parameters and breast cancer–specific survival in patients treated with cytotoxic drugs. Information on chemotherapy treatment in noncarriers was available for 26 *BRCA1* methylated tumors and 857 unmethylated tumors.

**Results:**

*BRCA1* was promoter methylated in 29 sporadic tumors or in 3.0% of cases (29 of 965), whereas none of the tumors derived from *BRCA* germline mutation carriers were promoter methylated. Important to note, patients with *BRCA1* promoter methylation receiving chemotherapeutic drug treatment show highly improved breast cancer–specific survival compared with unmethylated controls (hazard ratio = 0.10, 95% confidence interval = 0.01 to 0.75, two-sided *P* = .02).

**Conclusions:**

*BRCA1* promoter methylation is predictive of improved disease outcome in patients receiving cyclophosphamide, methotrexate, and fluorouracil drug treatment. Our results support the use of markers indicative of “BRCAness” in sporadic breast cancers to identify patients that are likely to benefit from the use of DNA-damaging agents.

Germline mutations in the breast cancer–susceptibility gene, Breast Cancer 1 gene (*BRCA1*), significantly increase the risk of developing breast and ovarian cancer, in addition to other forms of cancer (1). The *BRCA1* protein product is involved in DNA double strand–break (DSB) repair by homologous recombination, a highly conserved error-free DNA–repair pathway that uses an intact sister chromatid in late S or G2 phases of the cell cycle for the repair (2).

Tumors lacking *BRCA1* or *BRCA2* are homologous recombination deficient (HRD) and are characterized by mutational signatures, including indels, rearrangements, and base substitutions (3,4). Based on these mutational signatures, a model, HRDetect, has been developed to predict the *BRCA1*/*BRCA2* deficiency or “BRCAness” of tumors (3).

Loss of the wild-type allele is seen in most tumors arising in *BRCA1* mutation carriers (5). These tumors have high HRDetect scores compared with those that do not show loss of heterozygosity at the *BRCA1* loci. Tumors without loss of heterozygosity have similar HRDetect scores as noncarriers.

Germline mutations in *BRCA* genes and somatic mutations in homologous recombination genes are associated with increased sensitivity to platinum chemotherapy and Poly ADP Ribose Polymerase (PARP) inhibitors in breast cancer (6–10) and ovarian cancer (11–15). These agents induce replication fork stalling, creating DNA substrates that are dependent on homologous recombination for replication restart and are essential for the survival of the cell (16).

*BRCA1* is sometimes inactivated in breast tumors by promoter methylation (17,18). *BRCA1* methylated tumors are associated with the basal-like or triple-negative subtype that is predominant in germline mutation carriers (19,20). Recently *BRCA1* methylated tumors were associated with mutational signatures characteristic of tumors arising in *BRCA1* germline mutation carriers (4,21).

It is currently unclear, however, whether *BRCA1* promoter methylation translates to clinical benefits from the use of DNA-damaging agents in patients. We therefore carried out a large retrospective study aimed at determining whether *BRCA1* methylation is associated with improved outcomes in survival among chemotherapy-treated patients.

## Methods

### Study Group

The study group consisted of 1031 patients (women) diagnosed between 1976 and 2007 previously screened for the local *BRCA1* c.5074G>A and *BRCA2* c.767-771delCAAAT–germline mutations (22,23). In addition to the two *BRCA* founder mutations, *BRCA2* c.767-771delCAAAT and the much rarer *BRCA1* c.5074G>A, the only other *BRCA* mutation of some frequency, c.9976A>T, is not found to be associated with risk of breast or ovarian cancer but rather risk of small cell lung cancer and squamous cell carcinoma of the skin (24). Out of the 1031 cases, 965 were sporadic, 61 were *BRCA2* germline mutation carriers, and five were *BRCA1* mutation carriers. DNA samples from these patients were extracted from FF tissue samples (n = 417) and adjacent normal breast tissues (n = 91) using a standard phenolchloroform (+ proteinase K) method. DNA derived from formalin-fixed and paraffin-embedded tumors (n = 615) was extracted by deparaffinization using Octane followed by two rounds of ethanol washes and then overnight incubation in digestion buffer (50 mM Tris pH 8.8, 1 mM EDTA and 0.5% Tween, proteinase K).

This work was carried out according to the permits from the Icelandic Data Protection Commission (2006050307) and Bioethics Committee (VSNb2006050001/03–16).

### DNA–Methylation Analysis

The EZ-96 DNA Methylation-Gold kit (Zymo Research; D5008) was used to carry out bisulfite conversion of DNA samples derived from tumor and normal breast tissues. Primer design was based on the PyroMark Assay Desing 2.0 software (Qiagen). Pre-PCR reactions, using the Immolase DNA polymerase (Bioline; Bio-21047), along with pyrosequencing (PyroMark Q24; Qiagen) were carried as previously described (25). Four CpG sites were analyzed at genetic positions: chr17: 43125409(GRCh38.p7), chr17: 43125411(GRCh38.p7), chr17: 43125419(GRCh38.p7), chr17: 43125427(GRCh38.p7).

The primers used for pre-PCR were as follows: Forward primer 5-GTAGGGGTTTAGTTATTTGAGAAATTTT-3; reverse biotinylated primer 5-TATCCCTCCCATCCTCTAATTATAC-3. The sequencing primer for the pyrosequencing reaction was as follows: 5-AGTTTTAATTTATTTGTAATTTT-3. Tumor samples were considered to be *BRCA1* methylated at median greater than 10% methylation across the four CpGs (median values).

### Clinicopathological Parameters and Treatment

Information on patient age, birth date, and date of diagnosis, tumor grade, staging, and chemotherapy treatment given at time of diagnosis were obtained from the nationwide Icelandic Cancer Registry (26,27). Estrogen-receptor (ER) status was based on tissue-microarray (TMA) analysis (n = 664) (20,26) and, where TMA data were not available, we used data derived from routine clinical diagnoses based on dextran-coated charcoal assay used in Iceland in the period 1981 to 1995 (n = 98).

### Statistical Analysis

The χ^2^ and Fisher exact contingency table tests were carried out using base functions in R. The Kaplan–Meier method was used for generating univariable survival curves and the log-rank test was used for estimating *P* values. Hazard ratios (HRs) were estimated by applying the Cox proportional hazards model using R (survival package). Here, BRCA1–methylation status represents the main variable, whereas year of birth, year of diagnosis, ER status, nodal status, and tumor size are introduced as adjustment covariates. Patients were followed from the date of diagnosis until death or date of last follow-up (December 31, 2016). The endpoint was breast cancer–specific survival, defined as the time from diagnosis to death from breast cancer, as registered on death certificates. Patients who died of causes other than breast cancer were censored at the date of death. The cox.zph function in the survival package for R was used to test the proportional hazards assumption for a Cox regression model fit. All tests were two-sided and a P value of less than .05 was considered statistically significant.

## Results

### The Incidence of *BRCA1* Methylation in Primary Breast Tumors and Normal Tissue

**Figure 1.**
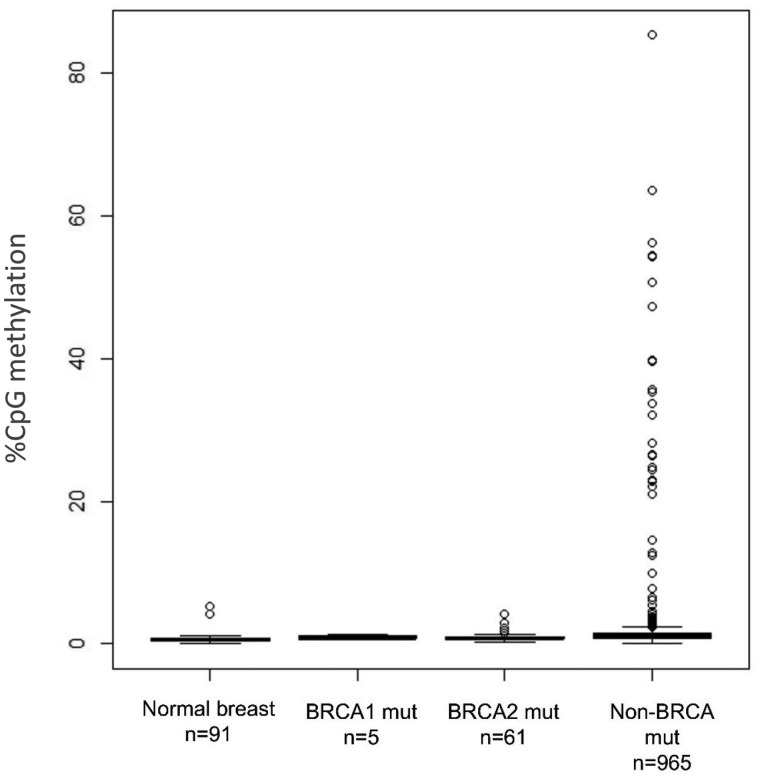
Box plot of BRCA1–promoter CpG methylation in normal breast tissue and tumors derived from carriers of germline mutations in BRCA1, BRCA2, and noncarriers.

We analyzed *BRCA1* promoter CpG methylation in 1031 primary tumors, along with 91 normal breast tissue samples derived from the same cohort. *BRCA1* methylation was identified in 29 tumors, none of which were derived from carries of germline mutations in either *BRCA1* (n = 5) or *BRCA2* (n = 61) (Figure 1). The frequency of *BRCA1* promoter methylation in non-*BRCA* mutated breast tumors is approximately 3% (29 out of 965; 3.0%).

### *BRCA1* Methylation in Relation to Clinicopathological Parameters

Information on ER status was available for 762 out of the 1031 tumors in our cohort; 700 out of the 762 tumors were derived from noncarriers (Table 1, A). We find that 90.5% of tumors with *BRCA1* promoter methylation are negative for ER, or 19 out of 21 (see Table 1, A). Similarly, tumors arising in *BRCA1* mutation carriers were exclusively ER negative (five out of five) as previously reported (20), and by TMA analysis, we find that the majority of *BRCA1* methylated tumors are of the basal-like subtype (eight out of 12; 67%) (see Table 1, B).

**Table 1.** CpG–promoter methylation of *BRCA1* gene analyzed with respect to clinicopathological parameters and breast cancer subtypes*

A) ER status with respect to BRCA1 methylation		
ER status	*BRCA1* unmethylated	*BRCA1* methylated
ER positive	526 (77.5%)	2 (9.5%)
ER negative	153 (22.5%)	19 (90.4%)
Total (N = 700)	679	21
		Fisher test, *P* < .0001

B) Breast cancers subtypes with respect to *BRCA1* methylation		

Subtype	*BRCA1* unmethylated	*BRCA1* methylated

LumA	78 (40%)	0
LumB	61 (31.3%)	1 (8.3%)
HER2	18 (9.2%)	0
Basal-like	34 (17.4%)	8 (66.7%)
5-negative (5NP)	4 (2.1%)	3 (25%)
Total (N = 207)	195	12
		χ^2^ = 39.0, *P* < .0001

C) Histological grade with respect to *BRCA1* methylation		

Histological grade	*BRCA1* unmethylated	*BRCA1* methylated

1+	104 (23.6%)	0
2+	187 (42.4%)	6 (31.6%)
3+	150 (34%)	13 (68.4%)
Total (N = 460)	441	19
		χ^2^ = 11.1, *P* = .0039

D) Tumor size with respect to BRCA1 methylation		

Tumor size	*BRCA1* unmethylated	*BRCA1* methylated

T1	462 (54.4%)	8 (30.8%)
T2	319 (37.6%)	16 (61.5%)
T3	54 (6.4%)	2 (7.7%)
T4	14 (1.6%)	0
Total (N = 875)	849	26
		χ^2^ = 6.9, *P* = .075

E) Nodal status with respect to *BRCA1* methylation		

Nodal status	*BRCA1* unmethylated	*BRCA1* methylated

Negative	422 (51.9%)	17 (65.4%)
Positive	391 (48.1%)	8 (30.6%)
Total (N = 839)	813	26
		Fisher test, *P* = .15

F) Age at diagnosis		

Age	*BRCA1* unmethylated	*BRCA1* methylated

≤55	439 (46.9%)	22 (75.9%)
>55	497 (53.1%)	7 (24.1%)
Total (N = 965)	936	29
		Fisher test, *P* = .002

* ER = estrogen receptor.

*BRCA1* promoter-methylated tumors are statistically significantly less differentiated based on histopathological grading (Χ^2^=11.1, *P* = .0039) (Table 1, C). However, no association was found with respect to clinical staging in terms of tumor size or nodal status (Table 1, D and E).

### The Effects of *BRCA1* Methylation with Respect to Responsiveness to Cytotoxic Treatment

**Figure 2.**
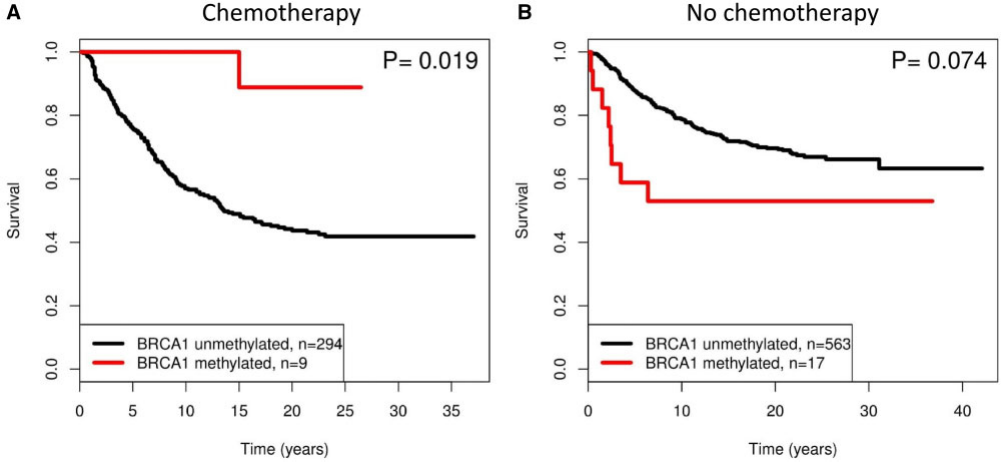
Breast cancer–specific survival time in patients treated **A**) with and **B**) without cytotoxic chemotherapy analyzed with respect to *BRCA1* methylation status. The *P* values shown reflect log-rank hypothesis testing for differences in survival times with respect to *BRCA1* methylation status.

To assess the association between *BRCA1* methylation and response to cytotoxic chemotherapy, we obtained information on chemotherapy and used time to patient death (breast cancer–specific survival) as a proxy for treatment response. In our cohort, cyclophosphamide, methotrexate, and fluorouracil (CMF) was the most commonly used cytotoxic treatment given at time of diagnosis, and nine of 29 patients with *BRCA1* methylated tumors received CMF treatment (Table 2). Information on chemotherapy treatment in noncarriers was available for 26 *BRCA1* methylated tumors and 857 unmethylated tumors. In noncarriers, patients with *BRCA1* methylated tumors show long-term survival following cytotoxic treatment (HR = 0.10, 95% CI = 0.014 to 0.751, *P* = .025) (Figure 2, A;Table 3). In contrast, nontreated patients with *BRCA1* methylation, compared with nontreated patients without *BRCA1* methylated tumors, showed similar time to breast cancer–specific death (Figure 2, B;Table 3). In comparing treated vs nontreated survival curves for non-*BRCA* mutated cases (Figure 2, A and B), there are notable differences, but these are likely explained by clinical presentation reflecting poor prognosis, and as a result, the treated group is biased toward reduced survival. This, however, does not affect our results, as we find improved survival for patients with BRCA1–methylated tumors after receiving cytotoxic treatment.

**Table 2.** Type of chemotherapeutic drugs used during treatment in the cohort of non-*BRCA* mutation carriers listed out with respect to *BRCA1* methylation status*

	*BRCA1* unmethylated	*BRCA1* methylated
CMF		
	242	9
		
Vincristine		
	97	2
		
Taxanes		
	9	0
		
Anthracycline		
	53	0
		
Any type		
	294	9

* CMF = cyclophosphamide, methotrexate, and fluorouracil.

**Table 3.** The effect of *BRCA1* methylation on time to breast cancer–specific death with and without chemotherapeutic treatment analyzed by multivariate Cox proportional hazards regression*

Treatment	HR (95% CI)	*P*
*BRCA1* methylation in nontreated patients (*n* = 391)	1.58 (0.52 to 4.80)	.416
*BRCA1* methylation in treated patients (*n* = 183)	0.10 (0.01 to 0.75)	.025

* Adjusted for year of diagnosis and birth, estrogen-receptor status, tumor size, and nodal status. CI = confidence interval; HR = hazard ratio.

In this cohort, nine patients with *BRCA1* methylated tumors received chemotherapeutic treatment with CMF (Table 2). However, patients with non-*BRCA1* methylated tumors received CMF or other treatments. By restricting our analysis to CMF–treated patients only, we find that the relation between *BRCA1* methylation and breast cancer–specific survival holds statistical significance (HR = 0.11, 95% CI = 0.014 to 0.81, *P* = .03).

## Discussion

In this article, we demonstrate that *BRCA1* promoter methylation, analyzed in a large cohort of 1031 primary breast tumors, predicts improved breast cancer–specific survival outcomes in patients treated with cytotoxic chemotherapy. No difference is seen in patients that did not receive a cytotoxic treatment, suggesting that *BRCA1* promoter methylation is a predictive factor for chemosensitivity but not a prognostic factor. Previous studies have shown that *BRCA1* mutation carriers are sensitive to treatment with platinum and PARP inhibitors. However, little is known about the effect of *BRCA1* methylation with respect to treatment based on DNA–damaging agents.

The classical CMF combination was the standard treatment for breast cancer patients until it was replaced by anthracyclines and taxanes as adjuvant treatment (28). Although it is less effective than anthracyclines and taxanes, large retrospective studies have shown a clear benefit of CMF, especially in triple-negative breast cancer (29–31). Although both methotrexate and fluorouracil (5-FU) are antimetabolites that block the synthesis of thymidine required for DNA synthesis, cyclophosphamide is an alkylating agent, and its cytotoxic metabolite phosphoramide mustard leads to inter- and intrastrand crosslinks in DNA causing replication fork stalling (32–34). Because of the importance of the DNA DSB repair machinery in DNA–replication restart, phosphoramide mustard treatment is likely to lead to synergistic lethality in BRCA–deficient cells. This mechanism of action is similar to that currently thought to underlie the effectiveness of PARP1 inhibitors and platinum drugs in killing *BRCA* deficient cells (16). The mechanism of PARP1 inhibition is however more complex than previously expected. In addition to generating persistent single-strand breaks leading to collapse of the DNA–replication fork, PARP1 becomes trapped on DNA when inhibited, forming a cytotoxic lesion leading to replication fork stalling (35). Recently PARP inhibitors were also shown to affect the recruitment of POLQ to DNA DSBs, inhibiting microhomology-mediated end joining or Alt-EJ pathway (36–38).

Recent data have shown that 40–70% of triple-negative breast cancer is HR deficient (3,4,39) and therefore likely to respond to agents causing DNA–replication fork stalling (40–43). Replication fork stalling possibly explains the efficacy of cyclophosphamide in triple-negative tumors (30,31), as these are likely to be HRD.

HRDetect is a recently introduced method that makes use of next-generation sequencing for identifying mutational signatures characteristic of tumors arising in *BRCA1* or *BRCA2* germline mutation carriers (3). The main idea behind HRDetect is to enable identification of tumors with defective homologous recombination repair. Indeed, Davies et al. found that tumors with *BRCA1* methylation were detected as HRD by HRDetect (3). However, it is unclear whether this finding translates to enhanced sensitivity of *BRCA1* methylated tumors to treatment with DNA–damaging agents. Our results suggest that, indeed, this does seem to be the case. It is important to note that HRDetect genomic signatures might still persist after restored homologous recombination function, especially in the metastatic setting. To discriminate between tumors that are truly HRD, functional methods such as RAD51 foci staining have been developed for clinical use to improve selection of patients sensitive to DNA–damaging agents (9,10).

Information on HRDetect scores was available for 24 tumors in our cohort, of which four were *BRCA1* methylated as previously described ([3]; Supplementary Figure 1, available online). The four *BRCA1* methylated tumors all show high HRDetect scores, comparable to values seen in *BRCA1* mutation carriers. Two of these four received CMF treatment, and both are still alive today, more than 18 years after their diagnosis.

Recently, in Tutt et al., advanced TNBC tumors where investigated for response to docetaxel and carboplatin, which revealed better response to docetaxel. These tumors, however, were pretreated with drugs that cause DNA lesions that require DNA DSB repair. As suggested by the authors, *BRCA1* methylation detected in the primary tumor may have been lost following this first treatment, thereby explaining the lack of response to carboplatin in the advanced tumors. Our study differs from that of Tutt et al. in that we investigate the response to first treatment in our patient cohort (8).

Of interest, we observe substantially lower frequency of *BRCA1* methylation than previously reported (17,18,44,45). The discrepancy likely reflects either smaller cohorts in previous studies, leading to a situation where the detection of a single methylated tumor greatly influences the frequency, or use of methods known to have a higher false-positive detection rate, compared with pyrosequencing, such as methylation-specific PCR. In our cohort, 3% of primary breast cancer samples are *BRCA1* methylated, which is in agreement with a recent study using data from The Cancer Genome Atlas (21).

Our cohort is based on DNA samples isolated from freshly frozen (FF) tumors (n = 417) and formalin-fixed paraffin-embedded (FFPE) tumors (n = 615). Previous studies have shown higher fraction of *BRCA1* methylation in FFPE samples (46). Our analysis, based on pyrosequencing, does not show statistically significant differences in frequency of *BRCA1* methylation in noncarriers (n = 965) between FFPE (14 out of 587; 2.4%) and FF (15 out of 378; 4.0%).

A limitation of this study lies in the low number of *BRCA1* methylated tumors detected in our cohort. However, we would like to point out that this is the largest cohort studied to date on *BRCA1* methylation. Another limitation lies in the use of time to breast cancer–specific death as a proxy for response to treatment.

In summary, we demonstrate that *BRCA1* methylation is an important predictive factor of chemosensitivity in breast cancer rather than being a prognostic factor. This provides support for the use of methods aimed at defining BRCAness to identify patients that will derive benefits from DNA–damaging chemotherapy treatment and, possibly, targeted therapy using PARP inhibitors.

## Funding

The authors would like to thank The Icelandic Research Fund (www.rannis.is) (14193–051 and 152077–051) and Gongum saman (www.gongumsaman.is) for funding.

## Notes

The funders had no role in the design of the study; the collection, analysis, and interpretation of the data; the writing of the manuscript; and the decision to submit the manuscript for publication.

The authors have no disclosures.

## Supplementary Material

pkz100_Supplementary_Data
