# Supplementary figures and images for: BRCA1 Promoter Methylation Status in 1031 Primary Breast Cancers Predicts Favorable Outcomes Following Chemotherapy
Source: JNCI Cancer Spectr. 2019 Dec 11;4(2):pkz100. doi: 10.1093/jncics/pkz100 (PMC7061679; doi:10.1093/jncics/pkz100)

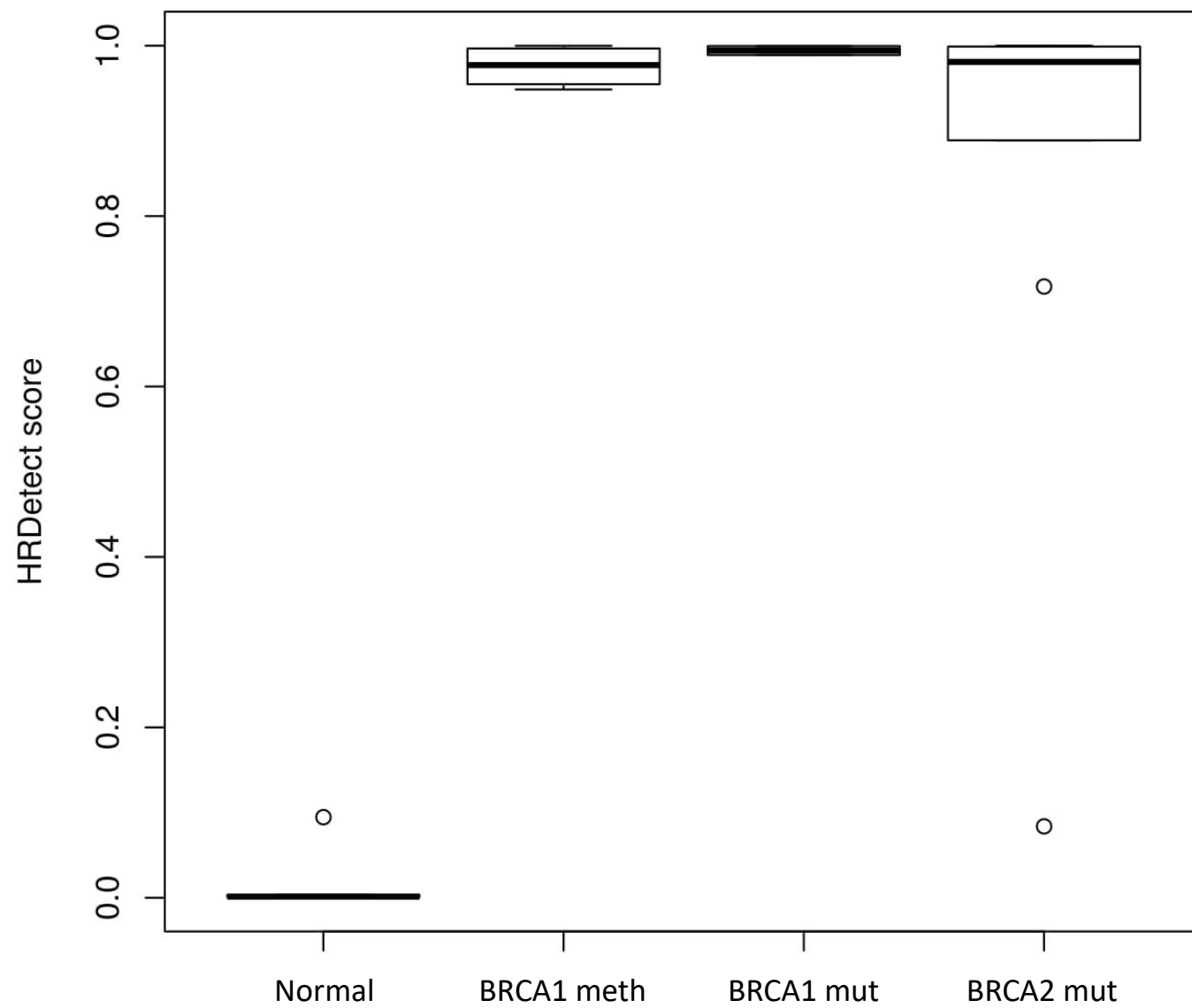

Supplement: pkz100_Supplementary_Data [file pkz100_supplementary_data.pdf]
